# New Guidelines of Pediatric Cardiac Implantable Electronic Devices: What Is Changing in Clinical Practice?

**DOI:** 10.3390/jcdd11040099

**Published:** 2024-03-27

**Authors:** Massimo Stefano Silvetti, Diego Colonna, Fulvio Gabbarini, Giulio Porcedda, Alessandro Rimini, Antonio D’Onofrio, Loira Leoni

**Affiliations:** 1 Paediatric Cardiology and Cardiac Arrhythmia/Syncope Unit, Bambino Gesù Children’s Hospital IRCCS, European Reference Network for Rare and Low Prevalence Complex Disease of the Heart (ERN GUARD-Heart), 00100 Rome, Italy; 2 Adult Congenital Heart Disease Unit, Monaldi Hospital, 80131 Naples, Italy; diego77c@libero.it; 3 Paediatric Cardiology and Adult Congenital Heart Disease Unit, Regina Margherita Hospital, 10126 Torino, Italy; gabbarini.f@gmail.com; 4 Paediatric Cardiology Unit, A. Meyer Children’s Hospital, 50139 Florence, Italy; giulio.porcedda@meyer.it; 5 Paediatric Cardiology Unit, G. Gaslini Children’s Hospital IRCCS, 16147 Genoa, Italy; alessandrorimini@gaslini.org; 6 Departmental Unit of Electrophysiology, Evaluation and Treatment of Arrhythmia, Monaldi Hospital, 80131 Naples, Italy; donofrioant1@gmail.com; 7 Cardiology Unit, Department of Cardio-Thoracic-Vascular Science and Public Health, Padua University Hospital (ERN GUARD-Heart), 35121 Padua, Italy; loira.leoni.1@unipd.it

**Keywords:** sudden cardiac death, tachyarrhythmia, bradyarrhythmia, pediatric age, defibrillator, cardiac pacing, guidelines

## Abstract

Guidelines are important tools to guide the diagnosis and treatment of patients to improve the decision-making process of health professionals. They are periodically updated according to new evidence. Four new Guidelines in 2021, 2022 and 2023 referred to pediatric pacing and defibrillation. There are some relevant changes in permanent pacing. In patients with atrioventricular block, the heart rate limit in which pacemaker implantation is recommended was decreased to reduce too-early device implantation. However, it was underlined that the heart rate criterion is not absolute, as signs or symptoms of hemodynamically not tolerated bradycardia may even occur at higher rates. In sinus node dysfunction, symptomatic bradycardia is the most relevant recommendation for pacing. Physiological pacing is increasingly used and recommended when the amount of ventricular pacing is presumed to be high. New recommendations suggest that loop recorders may guide the management of inherited arrhythmia syndromes and may be useful for severe but not frequent palpitations. Regarding defibrillator implantation, the main changes are in primary prevention recommendations. In hypertrophic cardiomyopathy, pediatric risk calculators have been included in the Guidelines. In dilated cardiomyopathy, due to the rarity of sudden cardiac death in pediatric age, low ejection fraction criteria were demoted to class II. In long QT syndrome, new criteria included severely prolonged QTc with different limits according to genotype, and some specific mutations. In arrhythmogenic cardiomyopathy, hemodynamically tolerated ventricular tachycardia and arrhythmic syncope were downgraded to class II recommendation. In conclusion, these new Guidelines aim to assess all aspects of cardiac implantable electronic devices and improve treatment strategies.

## 1. Introduction

The first Guidelines about PM implantation were published in 1984 [1]. Between 2008 and 2018, many documents have been published from the main Cardiology and Arrhythmia European and American Societies: the European Society of Cardiology (ESC), the European Heart Rhythm Association (EHRA), the Association for European Paediatric and Congenital Cardiology (AEPC), the Heart Rhythm Society (HRS), the American College of Cardiology (ACC) and the American Heart Association (AHA) [2,3,4,5,6,7,8,9,10,11,12,13,14,15,16,17,18]. They usually referred to adult patients with short paragraphs for pediatric patients. Indeed, there was a lack of specific Guidelines for pediatric cardiac pacing, as those published by the Pediatric and Congenital Electrophysiology Society (PACES) in 2012 on pediatric Wolff Parkinson White syndrome [19], by the AEPC/EHRA/ESC on arrhythmias in congenital heart disease (CHD) patients [20] and on pediatric tachyarrhythmias treatment [21,22]. In 2021–2023, four new documents were published: the 2021 PACES Consensus Statement on indication and management of pediatric Cardiovascular Implantable Electronic Devices (CIEDs) [23], the 2021 ESC Guidelines on cardiac pacing and cardiac resynchronization therapy [24], the 2022 ESC Guidelines for the management of ventricular arrhythmias (VA) and the prevention of sudden cardiac death (SCD) [25] and the 2023 HRS Guidelines on physiologic pacing [26]. The definitions of Class of Recommendation (COR) and Level of Evidence (LOE), present in all Guidelines, are reported in Table 1. The aims of this paper are to show the new Guidelines altogether to facilitate the approach for all health practitioners involved in pediatric and adult congenital heart disease (CHD) patients care and to show the most relevant changes that will influence clinical practice. 

## 2. Methods

We reviewed the four new Guidelines documents [23,24,25,26] and separated out and summarized recommendations for pediatric pacemaker (PM) and defibrillator (ICD) implantation classified in different diagnoses. The significant changes compared to previously published Guidelines have been highlighted. 

## 3. Relevant Sections

### 3.1. New Guidelines

#### 3.1.1. 2021 PACES Expert Consensus Statement [23]

This Consensus Statement was written with the cooperation of the main American (HRS, ACC, AHA), European (AEPC), Far East and Latin American Societies. The document was reviewed by a member of each society. It gives recommendations (COR) on the implantation and management of CIED: PM, ICD and Loop Recorder (Implantable Cardiac Monitor, ICM). It includes 130 recommendations about all pediatric arrhythmias, management of devices and complications. In the Statement, 28% are new recommendations, 12% are changes of COR, mainly of one level, for example from COR II a to II b, and 6% are changes of recommendations.

#### 3.1.2. 2021 ESC Guidelines [24]

They represent the official position of the ESC about cardiac pacing and cardiac resynchronization therapy. Similar to previous documents, they refer mainly to adult patients. Pediatric Guidelines are limited to a small paragraph about congenital isolated complete atrioventricular block (CCAVB), postoperative bifascicular block associated to transient complete AVB and sinus node dysfunction (SND).

#### 3.1.3. 2022 ESC Guidelines [25]

This document was developed by the ESC task force for the management of patients with VA and the prevention of SCD and presents an update of the 2015 ESC Guidelines [13]. It was endorsed by the AEPC.

#### 3.1.4. 2023 HRS Guidelines [26]

This is a clinical practice guideline of the HRS written with the cooperation of the main North American (HRS, ACC, AHA, PACES, Heart Failure Society of America, the International Society of Holter and Noninvasive Electrocardiology, ISHNE), Latin American and Asia-Pacific Societies. It provides recommendations for patients who require pacing or are at risk of heart failure. 

### 3.2. Pacing

Cardiac pacing indications according to arrhythmias and heart disease are reported in Table 2.

#### 3.2.1. Sinus Node Dysfunction (SND)

Isolated SND does not increase the risk of sudden death in patients with idiopathic SND. Therefore, in the three main PACES recommendations (COR I and II) for PM implantation [23], symptoms related to bradycardia or pauses are required. On the contrary, COR III indications refer to asymptomatic patients. However, symptoms may be likely attributable to bradycardia without conclusive evidence. In this case, a new indication was added (COR II b). Heart rate (HR) criteria remain only in SND associated with CHD: bradycardia < 40 bpm [23].

The ESC 2021 Guidelines consider only SND in patients with complex CHD and asymptomatic bradycardia (rest and awake HR < 40 bpm and pauses > 3 s.) with a COR II b. Bradycardia-related symptoms are not mentioned [24]. 

In SND, atrial pacing is better than ventricular pacing. Rate-responsive sensors will produce more physiologic stimulation [27,28]. In the PACES Statement, atrial or dual-chamber PM is recommended. However, as complications are mostly lead-related in pediatric age, fewer leads mean fewer future complications [29]. Moreover, the increased bulk in the PM pocket due to the larger dual-chamber PM and the second lead may favor local complications (erosion, infections). Single-chamber atrial pacemakers, either with transvenous or epicardial systems, are often adequate. Transvenous systems can be successfully upgraded afterward. 

#### 3.2.2. Isolated Congenital Advanced or Complete Atrioventricular Block (CCAVB) 

When there are risk factors for syncope or SCD (symptomatic bradycardia, broad QRS escape rhythm, complex ventricular ectopy, left ventricular, LV, dysfunction), PM implantation has a COR I. Mean HR is a COR I indication in infants and neonates, although the limit was reduced to 50 bpm instead of 55 bpm [6,7] to avoid too-early implantation. However, the HR is not the only factor to consider, as bradycardia and low cardiac output-related symptoms may also occur at higher HR. Risk/benefits of permanent pacing should be considered in children > 1 year, and HR criteria (<50 bpm or prolonged pauses), shifted from class I [2] to II a, as in the 2012 Guidelines [7]. A new recommendation, II a, is LV dilatation (Z score ≥ 3) associated with ventricular dysfunction or mitral regurgitation, risk factors for adult cardiovascular mortality [30]. The recommendation for PM implantation in adolescents without risk factors remained in COR II b, as in prior Guidelines [6,7]. Actually, benefits (prevention of syncope, heart failure, SCD) and risks (multiple PM and lead revisions, pacing-induced LV dysfunction) have to be considered. On the contrary, in asymptomatic adults with CCAVB, the recommendation was in COR II a in the 2018 Guidelines [2].

The ESC 2021 Guidelines recommended permanent pacing in CCAVB or advanced AVB when one of the following risk factors is present: symptoms, pauses longer than three times in the preceding cycle, broad QRS escape rhythm, prolonged QT interval, complex ventricular ectopy and mean daytime HR < 50 bpm. When risk factors are not present, COR is II b [24].

#### 3.2.3. Postoperative Block

In CHD patients, postoperative block occurs in 1–8% of patients [23,31,32]. Complete or advanced II degree postoperative AV block that persists 7–10 days after surgery is a class I indication [23]. This timing has been increased from >72 h in adults with CHD [2] or 7 days in pediatric patients [7] because spontaneous recovery of AV conduction increases from ≥85% at 7 days to ≥95% at 10 days [31,33]. However, in patients with transient postoperative advanced II or III degree AVB, there is a small risk of late-onset AVB that can occur months or years later. Postoperative bifascicular block (absent pre-surgery) may be a risk factor for this complication. When a late-onset III degree block or advanced II degree AV block occurs, especially when there is a history of transient AVB, the new recommendation for PM implantation is class I. Unexplained syncope in patients with previous transient postoperative AVB has a class II b recommendation. This recommendation was in class II a in the presence of residual bifascicular block [7]. The new recommendations in class II b are PM implantation before the limit of 7 days when AVB is not expected to resolve due to extensive injury to the conduction system; and transient advanced postoperative II or III degree AVB in selected patients predisposed to progressive conduction system abnormalities (AV discordance, septal AV defects, heterotaxy syndromes) [23]. The ESC 2021 Guidelines recommend (COR II b) PM implantation in postoperative bifascicular AVB associated with transient CAVB [24].

In the next sections about pacing in specific situations, we refer only to the PACES 2021 Consensus Statement [23], unless indicated.

#### 3.2.4. Other AVBs

This new section includes AVBs recognized during late childhood and adolescence. These AVBs may be congenital, acquired (infiltrative or inflammatory causes), idiopathic or paroxysmal [34,35]. All recommendations are new. Idiopathic symptomatic advanced II or III degree AVB not attributable to reversible causes is in class I. Class II a is for an AVB of any degree that progresses to an advanced II or III degree during exercise. In this case, conduction abnormality within the His-Purkinje system may be suspected, and prognosis without pacing is usually poor [7]. Intermittent advanced II or III degree AVB not attributable to reversible causes associated with minimal symptoms otherwise unexplained has a COR II b [23]. 

#### 3.2.5. CHD

Besides the same recommendations for patients with a structurally normal heart, specific considerations for patients with CHD are given in this section. Loss of vascular access to right heart chambers and persistent right to left shunt require epicardial pacing systems (COR III for endocardial leads). CCAVBs in neonates/infants with complex CHD have a COR I when there is hemodynamic compromise or mean HR is <60–70 bpm. Previous Guidelines required mean HR < 70 bpm [7]. As in CCAVB, the heart rate limit was decreased to avoid unnecessary early implantation. 

Class II a recommendations refer to antitachycardia pacing in CHD patients with recurrent intra-atrial reentrant tachycardia in whom ablation or medications were ineffective or not acceptable treatment [36,37]; atrial or dual-chamber pacing in patients with complex CHD and with impaired hemodynamic caused by sinus bradycardia or loss of AV synchrony as in single-ventricle physiology [28,38]. Class II recommendations apply to patients with SND, CHD and mean awake resting HR < 40 bpm and/or prolonged pauses: COR II a in the presence of a complex CHD; COR II b in the presence of a moderate CHD [23]. 

#### 3.2.6. Bradyarrhythmias Post-Cardiac Transplantation

In this section, the new recommendation is for PM implantation in any degree of AVB due to graft vasculopathy (COR II b) [39]. Persistent symptomatic bradycardia has Class I indication. 

#### 3.2.7. Neuromuscular Disease and Other Progressive Cardiac Conduction Disease

A new recommendation (COR II b) is PM implantation in patients with any progressive cardiac conduction disease at risk of rapid deterioration of AVN function even in the presence of normal AV function after considering patient age, size, etc. [40,41].

Changes of recommendation from the 2018 Guidelines [2] refer to Kearns-Sayre syndrome, myotonic dystrophy type 1, Lamin A/C mutation, including limb-girdle and Emery-Dreifuss dystrophies. Kearns-Sayre syndrome patients with any degree of AVB and/or conduction abnormality moved from Class IIa to Class I due to the unpredictable progression of conduction disease [42]. A PR interval > 240 ms with intraventricular conduction delay/left bundle branch block is a class II a recommendation for PM implantation in dystrophic/lamin A/C mutation patients. In these patients requiring pacing, a primary prevention ICD implantation may also be considered due to the malignant ventricular arrhythmias risk.

#### 3.2.8. Neurocardiogenic Syncope

Severe breath-holding spells with documented cardioinhibitory response and prolonged syncopal episodes, post-anoxic seizures and other bradycardia-induced symptoms moved to Class II a from II b [11]. On the contrary, cardioinhibitory syncope causing bradycardia or asystole, in which other treatments failed, has a COR II b. Pallid breath-holding syncope heals spontaneously, although some patients will later develop neurocardiogenic syndrome. Likewise, neurocardiogenic syncope often ends spontaneously during adolescence and young adulthood age. Therefore, pacing outcomes should be balanced against the complications of chronic pacing. Pacing is not indicated (COR III) in tilt-test-only induced cardioinhibitory syncope and in hypotensive syncope [23]. 

The recommendations for pediatric neurocardiogenic cardioinhibitory syncope differ from those of patients > 40 years of age in whom pacing is a Class II a recommendation [43]. Single-chamber pacing with hysteresis and dual-chamber pacing with rate drop response seem both effective for syncope and seizure prevention [44]. However, long-term pacing outcomes of this population are not reported. 

#### 3.2.9. PM Implantation in Channelopathies

**Figure 1:**
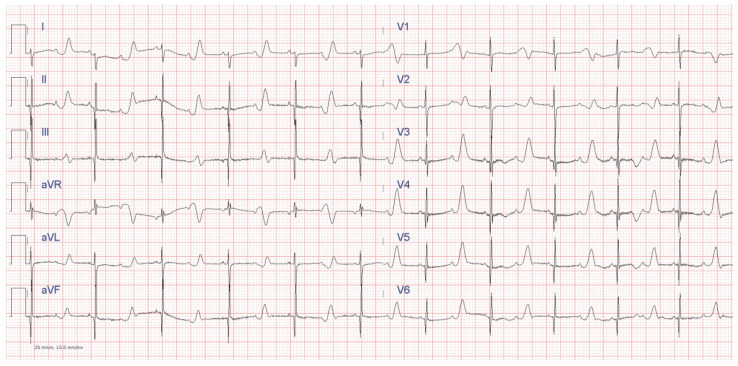
ECG of a newborn with Timothy Syndrome (LQTS 8), with severely prolonged QT and functional 2:1 AVB.

New recommendations in Class II b are given for LQTS and functional 2:1 AVB (Figure 1) or for channelopathies in which a faster HR may decrease arrhythmias burden and bradycardia-mediated symptoms. There is also a Class III recommendation for atrial-only pacing in atrial standstill (risk of pacing defects/failures). Ventricular pacing is the alternative.

### 3.3. Implantable Cardiac Monitor (ICM)

Modern ICMs have a documented diagnostic yield of around 60%. It is higher in symptomatic patients (89%) and in patients without structural heart disease (71%) [45]. The Class I recommendation is referred to patients with syncope and high-risk criteria when a thorough evaluation does not reveal the cause of syncope and does not lead to conventional indications for PM or ICD. Moreover, there are three new recommendations: ICM implantation is recommended (COR II a) to guide management of patients with channelopathies or structural heart disease and relevant arrhythmias; ICM implantation is recommended (COR II b) in patients with infrequent palpitations when other monitoring methods have been not effective (in case of palpitations a yield of 100% has been demonstrated [46]); finally, ICM implantation is recommended (COR II b) to detect subclinical arrhythmias in patients with channelopathies or other heart diseases [23,47]. 

### 3.4. Physiologic Pacing

In pediatric permanent pacing, besides anatomical (including surgical repair, body, vessels and heart chamber dimensions) and technical issues, the main concerns are the lifelong pacing duration and the risk of pacing-induced ventricular dysfunction [48,49,50]. Therefore, it is advised to implant ventricular leads at sites that optimize ventricular electrical activation and contractility, which are often patient-specific [51,52,53]. As a matter of fact, a site that is optimal for all patients has not been found. The new HRS Guidelines on physiological pacing [26] (CPP, cardiac physiologic pacing, and CSP, conduction system pacing) [54] aim to prevent pacing-induced LV systolic dysfunction. RV lead implant sites close to the normal conduction system (His bundle area, inflow, mid-septum and left bundle branch, LBB, area) seem to preserve or improve contractility [55,56,57]. However, His bundle pacing (HBP) may result in high thresholds, leading to dislodgements and failures [26]. Therefore, in patients with CCAVB requiring permanent pacing, CSP or pacing close to the conduction system has a Class II a recommendation. With epicardial pacing, left ventricular apex is the preferred site (COR II a), and results are good [55,58,59]. 

When left ventricular dysfunction or symptoms of heart failure occur during chronic pacing, cardiac resynchronization therapy (CRT) is recommended (class II a). On the other hand, when LV dysfunction/congestive heart failure occurs in non-paced patients with AVB requiring PM, physiologic pacing has a class II b recommendation. Preliminary data seem to suggest that CPP/CSP may be an alternative to CRT in right ventricular (RV) epicardial pacing patients with signs of impaired systolic function: CPP/CSP seems to improve electromechanical function narrowing QRS and improving EF [55]. Children with CCAVB and normal ventricular function often show high values of stroke volume and EF to counterbalance low HR and maintain good cardiac output. In these patients, after pacing, LV end-diastolic dimension z-score decreased, and EF remained within normal limits [60].

**Figure 2:**
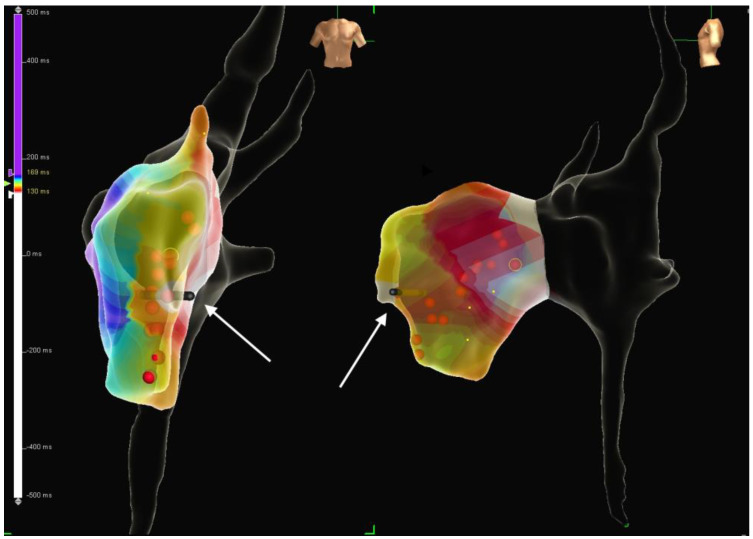
Three–dimensional electroanatomic mapping system showing the position of the pacing lead implanted in the subpulmonary LV in a patient with congenitally corrected transposition of the great arteries (CCTGA) and dextroposition of the heart. Red dots show the LBB potential recording sites; the white arrows show the lead tip. Abbreviations as in text.

In CHD and AVB patients, class II a recommendations for physiological pacing are: CRT in the presence of systemic left ventricle dyssynchrony (QRS duration > 3 Z score) and EF < 45% [61,62]; apical pacing in single ventricle [63]; fusion-based pacing in RV dysfunction and right bundle branch block (RBBB) [64]; CSP in congenitally corrected transposition of the great arteries (CCTGA) with AVB [65,66,67,68]. In CCTGA, the subpulmonary ventricle is the anatomical LV, and the conduction system found on the endocardial subpulmonary ventricle surface is the LBB, with its anterior and posterior fascicles. The distal His bundle is elongated and anteriorly located, and it courses below the pulmonary valve. Therefore, physiologic pacing requires non-selective HBP or RBB area pacing on the deep septum or endocardial surface of the left-sided RV. The right bundle is a thin structure and usually courses superiorly to LBB; therefore, RBB area pacing would be performed on the septum above the recorded course of the LBB (Figure 2). The RV activation time is measured in V5 or V6.

Pediatric physiologic pacing has been accomplished with stylet-driven leads [55] and with lumenless leads [54,69,70].

### 3.5. Implantable Defibrillator (ICD)

Recommendations for ICD implantation are given in the PACES Consensus Statement [23] and in the ESC Guidelines [25]. Generally speaking, secondary prevention is an easy choice for physicians. There are no doubts that a resuscitated SCD in whatever cardiac disease (a possible exception is CPVT, see below) has to be implanted with an ICD unless life expectancy is less than 1 year. Main problems arise with primary prevention. Physicians have to recommend an ICD implantation in patients who are at risk of SCD but who are generally asymptomatic, and in some inherited or acquired cardiac disease, apparently healthy. For this difficult purpose, Guidelines are most useful. Further, risk calculators have been developed and are often inserted in new Guidelines. Shared decisions among physicians, families and patients are required before ICD implantation. Primary prevention recommendations are reported in Table 3. 

#### 3.5.1. CHD

In the PACES Consensus Statement, secondary prevention is in class I in the case of hemodynamically unstable sustained VT (SVT), although ablation or surgical repair may be alternatives in selected cases [23]. In the ESC Guidelines, for all CHD with non-tolerated VT or cardiac arrest caused by VF, secondary prevention is addressed in class I after having excluded reversible causes [25]. In patients with tetralogy of Fallot, preserved biventricular function and symptomatic SMVT, catheter or surgical ablation may be alternatives to ICD [25].

The PACES and ESC primary prevention recommendations are reported in Table 3. The only COR I recommendation for primary prevention regards adult CHD with systemic LV, symptomatic heart failure (NYHA II/III) and LVEF ≤ 35%, despite ≥ 3 months of optimized medical therapy (OMT) according to the ESC Guidelines [25].

#### 3.5.2. Cardiomyopathies (CMPs)

In both documents, there is general agreement about secondary prevention. 

The PACES recommendation is more specific for pediatric age. Cardiac arrest and SVT, hemodinamically tolerated or not, are in class I for dilated (DCM) and hypertrophic cardiomyopathies (HCMs). On the contrary, in arrhythmogenic cardiomyopathy (ACM), class I recommendation is reserved to cardiac arrest and not tolerated SVT [23]. 

In the ESC Guidelines, hemodynamically not tolerated VT is a class I recommendation, while hemodynamically tolerated VT is class II a recommendation for ICD in the three main CMPs [25]. 

Primary prevention has more complex recommendations, as shown in Table 3. 

Following previous American Guidelines, the PACES Statement considered risk factors (RF) for SCD in HCM: unexplained syncope, NSVT, family history of early SCD and HCM-related, massive left ventricular hypertrophy (LVH) [23]. Primary prevention interventions in HCM identified extreme LVH as the most common marker, alone or in combination. ICD interventions occurred in the same proportion (14%) for patients who underwent implantation for 1, 2 and ≥3 risk factors, and the annual intervention rate for secondary prevention was 13%, while it was 3% for primary prevention [71]. Most of the pediatric studies about ICD in cardiomyopathies have focused on HCM [72,73,74,75].

The European Guidelines do not include familial history of SCD related to HCM in the RFs. They apply the HCM-Risk calculator and the Risk Kids score [76] for patients > 16 years or <16 years, respectively [25]. 

A risk calculator was also developed in adult ACM patients [77]. Although the entirely subcutaneous ICD system (S-ICD) is effective and safe in ACM [78,79,80], the ESC Guidelines suggest that antitachycardia pacing-enabled devices for sustained monomorphic VT (SMVT) should be considered (COR II a) [25].

In pediatric dilated cardiomyopathy (DCM), primary prevention recommendations in the presence of syncope or LVEF ≤ 35% and OMT moved to COR II b rather than COR I [12,13]. The reason was the low incidence of SCD in pediatric DCM and the risks of ICD [23]. 

In the ESC Guidelines, symptomatic heart failure (NYHA class II–III) with LVEF ≤ 35% after three months of OMT is a class II a recommendation, as well as in Lamin A/C mutations. In Lamin mutations, independent risk factors for ventricular tachyarrhythmias were non-sustained VT (NSVT), male sex, LVEF < 45% and non-missense mutation. Recently, an adult risk calculator [81] has been developed and included in the ESC Guidelines. In patients with a 5-year estimated risk ≥ 10% and with a manifest cardiac phenotype (Table 3), ICD should be considered [25]. 

In patients with LV non-compaction cardiomyopathy, a primary prevention ICD implantation should follow the DCM recommendations [25].

Data about the ICD use in restrictive cardiomyopathy (RCM) patients are limited. ICD recommendations usually follow the HCM Guidelines, although patients with RCM showing heart failure or unexplained syncope may appropriately receive an ICD when the transplant option is not immediate [23].

#### 3.5.3. Myocarditis

VA may occur in acute or chronic myocarditis [82]. Recommendations refer only to the ESC Guidelines [25]. In the acute phase, patients with VF or not hemodynamically tolerated SVT should be considered for an ICD (II a). In chronic myocarditis, ICD is recommended in patients with hemodynamically not tolerated SMVT (COR I) and should be considered in those with hemodynamically tolerated SMVT (COR II a). 

#### 3.5.4. Channelopathies

##### Long QT Syndrome (LQTS)

Secondary prevention is a class I recommendation [23,25]. In primary prevention, a new recommendation from PACES (COR II b) includes some clinical risk factors: severely prolonged QTc (QTc > 550 in any patient or >500 ms according to genotype), specific genotypes as Jerwell-Lange-Nielsen, Timothy syndrome [83], calmodulinopathies and other phenotype risk factors. They include onset of symptoms <10 years of age, prior SCA and recurrent syncope. Infants with bradycardia and functional 2:1 AVB (Figure 1) have a significant risk [23]. 

The ESC Guidelines recommend ICD in class I in symptomatic patients on beta blockers (BBs) and genotype-specific therapy. Left cardiac sympathetic denervation (LCSD) is recommended in symptomatic patients with multiple ICD shocks, syncope due to VA, and when ICD is contraindicated or refused. ICD or LCSD should be recommended (Class II a) in symptomatic patients when BBs and other genotype-specific therapies are not tolerated or contraindicated. ICD may be considered in high-risk asymptomatic patients (according to 1-2-3-LQTS risk calculator) [84] as an adjunct to genotype-specific therapy [25].

New criteria are given for diagnosis and treatment of Andsersen-Tawil syndrome. This diagnosis can be made in the presence of ≥2 factors (prominent U waves with/without QTc prolongation; bidirectional polymorphous premature ventricular complex (PVC)/VT; dysmorphic features; periodic paralysis; pathogenic loss of function mutation of KCNJ2 gene). ICD implantation is recommended (COR I) in aborted SCA or non-tolerated sustained VT (SVT); it may be considered in patients with unexplained syncope or with tolerated SVT (COR II b). ICM should be considered in the presence of unexplained syncope (COR II a) [25]. 

##### Short QT Syndrome (SQTS)

New diagnostic criteria are given: QTc ≤ 320 ms; QTc ≤ 360 ms and arrhythmic syncope (II a); QTc ≤ 360 ms and a family history of SCD < 40 years of age (II b) [25]. In these patients, an ICD is recommended in secondary prevention. A new recommendation for primary prevention is for arrhythmic syncope (COR II a). An ICM should be implanted (COR II a) in young patients with SQTS [25].

##### Early Repolarization Syndrome (ERS)

New recommendations for ICD implantation are given (Table 3). ICM should be considered (COR II a, LOE C) in individuals with early repolarization patterns (ERPs) and at least one risk featured (Table 3) or arrhythmic syncope [25].

##### Catecholaminergic Polymorphic Ventricular Tachycardia (CPVT)

The PACES and ESC Guidelines recommend secondary prevention (class I) in CPVT patients with CA or arrhythmic syncope despite maximally tolerated therapy with beta blockers (BBs) and flecainide and/or left cardiac sympathetic denervation (LCSD) [23,25]. However, when aborted SCA was the initial presentation of CPVT, in some studies, ICD was not associated with improved survival [85,86]; therefore, in this case, ICD in association with drug, and with or without LCSD, now has a COR II a, and pharmacologic therapy and/or LCSD without ICD may be considered as an alternative strategy [23]. However, the ESC Guidelines suggest caution to downgrade ICD implantation in patients with CPVT who survived CA [25]. 

Polymorphic/bidirectional VTs are downgraded to class II b because of the automaticity mechanism of these arrhythmias [23]. Inappropriate painful shocks might increase the sympathetic tone and trigger further ventricular arrhythmias, leading to electrical storms and death. Therefore, ICDs should be programmed with high-rate cut-offs and long delays before shock delivery in the VF zone only [12,13,14,23,25,85].

The ESC Guidelines recommend (class II a) ICD implantation in patients with arrhythmic syncope and/or documented bidirectional or polymorphous VT on BBs and flecainide therapy at maximal tolerated doses. LCSD has a similar recommendation when BBs plus flecainide therapy is ineffective, not tolerated or contraindicated [25].

##### Brugada Syndrome

New diagnostic criteria are reported in the ESC Guidelines. COR IIa: type 1 induced Brugada pattern and ≥1 RF (arrhythmic syncope or nocturnal agonal respiration; family history of BrS; family history of SD < 45 years). COR II b: induced type 1 Brugada pattern and no other heart disease [25]. 

Secondary prevention is reserved (class I) for both Guidelines after a SCA and/or sustained spontaneous VT. In primary prevention, recent syncope due to suspected VA changed from class I [12] to class II a in the presence of spontaneous pattern to class II b in the presence of induced pattern [23]. 

An ICM is recommended (COR II a) in Brugada patients and unexplained syncope [25]. In pediatric patients, ICM can identify occult arrhythmias and the occurrence of arrhythmias during symptoms [87,88,89].

## 4. Discussion

Although recognizing the efficacy of current pacing technology and practice, the new Guidelines underline the concept that, whenever possible, permanent pacing should be delayed to decrease the risk of complications. The evaluation of benefits/risks should always be performed to decrease over- and under-treatment. For this reason, the most important changes in pacing recommendation compared to previously published Guidelines are the lower HR limit to recommend PM implantation in CCAVB and in AVB associated with CHD and the longer waiting time in postoperative AVB. On the contrary, in SND, the presence of symptoms is the main risk factor. Rate limits were only reported in SND associated with moderate or complex CHD (COR II b). The main differences between the two Guidelines on cardiac pacing are the long QT in the escape rhythm considered among risk factors (COR I for the ESC Guidelines) [24] and the AVB that progresses to advanced II–III degree with exercise (COR II a for the PACES statement) [23]. Regarding ICD implantation, the main changes are in primary prevention recommendations. In HCM, pediatric risk calculators have been included in the European Guidelines [25]. In DCM, due to the rarity of SCD in pediatric age, low ejection fraction criteria were demoted to Class II [23,25]. The presence of late gadolinium enhancement in cardiac magnetic resonance is considered as a new risk factor for HCM [23,25] and DCM [25]. Also, pathogenic gene mutations have been included among risk factors for ACM and LQTS [23] and for DCM [25]. 

When the decision of device implantation is taken, the second decision is what pacing system should be implanted. In the nineties, before steroid-eluting leads, endocardial systems performed much better than epicardial ones, and besides epicardial systems, transvenous pacing systems were implanted even in infants and small children [90,91,92]. Since the introduction of steroid-eluting leads [29,93,94,95,96,97,98], both transvenous/endocardial and epicardial systems have been effective, although the latter, in all reports, showed the worst outcome due to a higher frequency of lead malfunction/failure/fracture. The higher frequency of epicardial lead failure is probably due to increased susceptibility to trauma and stress imposed on leads by thoraco-abdominal movements. Therefore, there are long-term follow-up data that give us information about chronic complications of both pacing systems [29,91,92,96,97,98,99]. Lead failure, venous occlusion, late dislodgement causing lead failure and abandonment and tricuspid valve damage have been frequently reported with endocardial pacing. Moreover, somatic growth may straighten, tension and stretch transvenous leads, a phenomenon highlighted by a taut appearance on chest X-ray, that may cause late lead dislodgement and failure [96]. A few techniques have been proposed to avoid lead traction and stretching and to maintain lead function until growth is completed: absorbable ligature [100], atrial loop for ventricular lead [92,101] and periodical lead advancement [99]. Despite initial favorable results, some concerns arose, and complications related to these techniques have been described [102]. 

In young patients, infection risk is relatively low, being around 1–5% of chronic leads [29,103]. 

Therefore, permanent pacing in neonates/infants should be performed with epicardial systems, while endocardial systems implantation should be delayed after a certain age and weight, considering the operator’s skills and experience. These limits can be reasonably set at 10–15 kg and around 3–4 years. Some centers delayed transvenous system implant until adolescence [97]. Pacemaker syndrome is very rare in the young. Therefore, children usually require only VVIR pacing [104,105], and dual-chamber systems can be implanted or upgraded later in adolescence/young adulthood (Table 4).

Another relatively new issue about pediatric pacing is the awareness of limiting radiation exposure during PM implantation. This is even more important in selective site or physiologic pacing, where more difficult procedures often require higher radiation doses. The use of three-dimensional (3-D) electroanatomic mapping systems (Figure 2) allowed a significant reduction in radiation doses [55,106,107]. However, some parts of the implantation procedure still require X-rays: venous angiography, although an echo-guided venous approach may be a reasonable alternative; metallic wire progression in the veins–heart chambers; screw-in lead implantation; the need to leave adequate lead slack or atrial loop for growth. Therefore, near-zero procedures are good results. 

The axillary vein approach in children is safe and effective [108]. It has been shown to be better than the subclavian approach by reducing the risk of lead fracture due to the subclavian crush syndrome, where there is mechanical entrapment of the lead between the costo-clavicular ligament and the subclavius muscle [109]. Cephalic vein cutdown is an alternative, although the small size of the vein limits its use in children. Moreover, sports with pronounced arm movements may increase the risk of late lead damage due to subclavian crush. Therefore, implantation on the contralateral side of the dominant arm with an axillary approach may improve the durability of the system and allow sports participation [110]. The PACES Consensus Statement pointed out that in patients with CIED, participation in exercise and sports is mainly based on the consideration of the diagnosis and physiology of the patient rather than the presence of the CIED [23].

Besides transvenous ICD systems, epicardial and subcutaneous ICDs are implanted. Epicardial devices are placed in an abdominal pocket, with epicardial leads for pacing and sensing, and defibrillation array/coils implanted in the subcutaneous tissue, pericardial or pleural space [111,112,113,114]. 

The efficacy of these systems is good, but complications are frequent. The most frequent complications are lead malfunctions, erosions/infections and vascular or valvular problems, similar to those of PM systems, although they occur more frequently due to the design and dimensions of leads and devices [115,116]. Moreover, inappropriate shocks are frequent. Abdominal defibrillator cans and subcutaneous coils can migrate because of somatic growth and change the electrical field to prevent correct defibrillation. Intra-pericardial leads and coil may cause strangulation. Further, infants and toddlers are particularly challenging cases [117]. New implantation techniques of epicardial systems, minimally invasive [118] or with pleural shock coils and the devices placed in a subcardiac, extrapericardial location, seemed to lower failures and complications [112,113,114]. However, such unusual device placement increases the risks of replacement procedures at the end of battery life.

The S-ICD, without transvenous, intracardiac and intrathoracic access, reduces the operative risks, lead complications and risks of endocarditis or sepsis and offers many advantages in growing patients. It preserves venous patency and tricuspid valve function. Strong indications for S-ICD implantation are young age, primary prevention, poor vascular access, previous system infection or high infection risk. Contraindications are: need for antibradycardia and/or antitachycardia pacing and failed screening. The rate of pediatric patients eligible at the screening test for S-ICD was around 80% [119]. Pediatric studies showed that appropriate shocks were delivered in 9–27% of patients, inappropriate shocks in 7–25%, and complications occurred in 4–27% of patients [119,120,121,122,123,124,125,126]. A multicenter European study, The Sidecar Project, reported rates of 17% for appropriate shocks, 13% for inappropriate shocks and 9% for complications. In this study, neither defibrillation failure nor lead malfunction occurred. The three-incision technique implantation procedure and a body mass index (BMI) < 20 were risk factors for complications requiring surgical revision [127]. This finding is related to the dimensions of the device, which are still too large for children. Technique improvement (two-incision procedure and intermuscular pocket) showed better results. Few studies compared the outcomes of S-ICD and transvenous ICD. There were no significant differences in the efficacy of both systems [128], while S-ICD showed less frequent lead-related complications and inappropriate shocks and more frequent pocket-related complications [129]. 

In conclusion, the indications for ICD implant in young patients should be (Table 4):

1. Epicardial ICD system with subcutaneous, pericardial or pleural shock coils should be implanted in infants and small children.
2. The implantation of an ICD with transvenous single lead and single coil seems to be the best choice in children weighting more than 30 kg.
3. Dual-chamber ICDs, unless strictly necessary, may be implanted after puberty.
4. S-ICD may be the preferred choice in young patients with a BMI > 20, unless contraindicated.

## 5. Conclusions

The new Guidelines along with technical improvements allow better treatments with CIED of the relatively small but certainly tricky and challenging population of pediatric patients. Furthermore, the knowledge of the long-term outcome of children should guide the operators to perform a safe and conservative approach early in childhood according to child dimension, diagnosis, anatomy and physical activity. More complex systems can be implanted later after puberty. 

There are current gaps in knowledge that limit societies’ ability to provide robust recommendations. All pediatric recommendations have LOE B, C (Table 1), as randomized studies (LOE A) are lacking. Many pediatric experiences came from single or few cases or from small series/cohorts. Consensus expert opinion papers are often relevant. Prospective studies and multicenter experiences, including larger cohorts and registries, may provide new and more robust data. 

## 6. Future Directions

New approaches to pacemakers or ICDs and lead placement are ongoing [130,131,132,133]. Leadless pacemakers are promising systems that should minimize complications related to lead and pocket [134,135], although the large size of venous introducers limits their pediatric use. Moreover, there are concerns about the unsolved issue of device removal at the end of battery life. The association of an S-ICD with a leadless PM will increase the indications of these two innovative systems. The single chamber extravascular ICD is another new system that may result in being useful in some children [136].

## Figures and Tables

**Table 1:** List of Class of Recommendation and Level of Evidence.

Class of Recommendation (COR)		
Class I	Benefits >>> Risks.	Procedures/treatments should be performed/are recommended
Class II a	Benefits >> Risks.	It is reasonable to perform procedure/treatment
Class II b	Benefits ≥ Risks.	Procedures/treatments may be performed/uncertain efficacy
Class III	Risks ≥ Benefits.	Procedures/treatments should not be performed/are not useful/may harm
Level of Evidence (LOE)		
LOE A	Data derived from multiple randomized clinical trials or meta-analyses	
LOE B	Evidence from non-randomized, observational studies or from registry (B-NR)	
LOE C	Limited evidence from observational studies or case series (C-LO) or Consensus expert opinion, case studies, standard of care (C-EO).	

**Table 2:** Recommendations for PM implantation in pediatric patients.

COR, LOE	ARRHYTHMIA, RECOMMENDATION	PREVIOUS COR
	AVB	
I LOE B	-CCAVB with symptomatic bradycardia or in the presence of risk factors: wide QRS escape rhythm, complex ventricular ectopy, ventricular dysfunction (PACES)	
I LOE C	-CCAVB or advanced AVB with one of the following risk factors: symptoms, pauses > 3 times the cycle length of the ventricular escape rhythm, wide QRS escape rhythm, long QT, complex ventricular ectopy, mean daytime HR < 50 bpm (ESC)	
I LOE C	-CCAVB in asymptomatic neonates/infants and mean HR ≤ 50 bpm (PACES)	<55 bpm [7]
I LOE C	-CCAVB in neonates/infants with complex CHD when bradycardia is associated with hemodynamic compromise or mean HR < 60–70 bpm (PACES)	<70 bpm [7]
I LOE C	-symptomatic patients with idiopathic advanced II or III AVB not attributable to reversible causes (PACES)	New
I LOE C	-Clinically significant pause-dependant VT or associated with severe bradycardia (reasonable alternative: ICD) (PACES)	
II a LOE B	-asymptomatic CCAVB patients > 1 year old and mean HR < 50 bpm or with prolonged pauses (PACES)	I [2]
II a LOE C	-CCAVB with LV dilatation (Z score ≥ 3) associated with significant mitral regurgitation or systolic ventricular dysfunction (PACES)	New
II a LOE C	-any degree of AVB that progresses to advanced II or to III with exercise without reversible causes (PACES)	New
II b LOE C	-CCAVB in asymptomatic adolescents with an acceptable HR and without risk factors (PACES)	II a [2]
II b LOE C	-CCAVB or high degree AVB without risk factors (ESC)	
II b LOE C	-intermittent advanced II or III AVB without reversible causes and associated with otherwise unexplained minimal symptoms (PACES)	New
III LOE C	-PM not indicated in asymptomatic I degree or II Mobitz type 1 AVB (PACES)	
	**POSTOPERATIVE AVB**	
I LOE B	-postop. advanced II or III that persists for at least 7–10 days after cardiac surgery (PACES)	>7 days [7] >72 h [2]
I LOE C	-late onset postop. advanced II or III AVB, especially when there is prior history of transient postop. AVB (PACES)	New
II b LOE C	-unexplained syncope if there is a history of transient postop. advanced II or III AVB (PACES)	II a [7]
II b LOE C	-postop. advanced II o III AVB < 7 days that is not expected to resolve due to extensive injury to conduction system (PACES)	New
II b LOE C	-transient advanced postop. II or III degree AVB in selected patients predisposed to progressive conduction system abnormalities (PACES)	New
II b LOE C	-persistent postop. bifascicular block associated with transient complete AVB (ESC)	
	**ISOLATED SND**	
I LOE B	-SND with symptoms correlated with age-inappropriate bradycardia (PACES)	
I LOE C	-symptomatic SND secondary to chronic medical therapy for which there is no alternative treatment (PACES)	
II a LOE C	-in patients with symptoms temporally associated with chronotropic incompetence (rate-responsive PM) (PACES)	
II B LOE C	-in SND with symptoms likely attributable to bradycardia or prolonged pauses without conclusive evidence correlating symptoms with bradycardia after a thorough evaluation (PACES)	New
III LOE C	-asymptomatic SND (PACES)	
III LOE C	-symptomatic SND due to a reversible cause (PACES)	
	**SND IN CHD**	
II a LOE B	-PM with antitachycardia pacing in patients with CHD and recurrent IART when catheter ablation or medication are ineffective or not acceptable treatments (PACES)	
II a LOE C	-in patients with CHD and impaired haemodynamics due to sinus bradycardia or loss of AV synchrony (PACES)	
II a LOE C	-in patients with brady-tachy syndrome and symptoms attributable to pauses for sudden-onset bradycardia (PACES)	
II a LOE C	-sinus or junctional bradycardia in complex CHD when mean awake resting HR is <40 bpm or with prolonged pauses (PACES)	
II b LOE C	-sinus or junctional bradycardia in simple or moderate CHD when mean awake resting HR is <40 bpm or with prolonged pauses (PACES)	
II b LOE C	-asymptomatic bradycardia with awake resting HR < 40 bpm or with pauses > 3 s in complex CHD (ESC)	

AVB: atrioventricular block, CHD: congenital heart disease, SND: sinus node dysfunction.

**Table 3:** Recommendations for ICD implantation for primary prevention of sudden cardiac death in pediatric cardiomyopathies, channelopathies and congenital heart diseases.

COR, LOE	Recommendations for ICD Implantation PACES 2021	Recommendations for ICD Implantation ESC 2022	Changes (PACES if Not Otherwise Specified)
**HCM**			
		ESC risk calculator in patients > 16 aa	
		Risk Kids score in patients < 16 aa	
II a LOE B	≥1 RF: Syncope, NSVT, familial history of SCD, severe LVH	-ESC risk calculator: ≥6%	≥2 RF [12]
		-ESC risk calculator: ≥4, <6% (in the presence of LGE > 15% LV mass, LVEF < 50%, exercise-related hypotension, apical aneurism)	NEW-ESC
		-Risk Kids score: ≥6%	NEW-ESC
II a LOE C		Hemodynamically tolerated SMVT	NEW-ESC
II b LOE B	HCM with only other RF:	-ESC risk calculator: ≥4, <6%	NEW-ESC
	LGE, systolic dysfunction	-ESC risk calculator: <4 (low risk) in the presence of LGE > 15% LV mass, LVEF < 50%, apical aneurism	
**ACM**			
		Definite ACM	
II a LOE B	Hemodynamicslly tolerated STV, arrhythmic syncope, LVEF ≤ 35%,	Arrhythmic syncope	I [12] NEW-ESC
II a LOE C		Severe LV or RV systolic dysfunction, moderate dysfunction with NSVT and SMVT inducibility	
II a LOE C		Symptomatic (palpitations, presyncope) patients with definite ACM, moderate RV or LV dysfunction, and either NSVT or inducibility of SMVT at EPS	NEW-ESC
IIa LOE C		Hemodynamically tolerated SMVT	
II b LOE C	ACM with + genetic and addictional RF		new
**DCM**			
II a LOE A		Symptomatic CHF (NYHA II-III) and LVEF ≤ 35% in OMT,	I [12,13]
		LMNA mutation (risk calculator score ≥ 10%, NSVT, LVEF < 50%, AV conduction delay)	
II a LOE C		-LVEF ≤ 50% with > 2 RF (LGE, SMVT inducibility at EPS, pathogenic mutations of LMNA, PLN, FLMC, RBM20 genes)	NEW-ESC
		-Patients with hemodynamically tolerated SMVT	NEW ESC
II b LOE C	Syncope, LVEF ≤ 35% with OMT		I [12]
**LQTS**			
I LOE B	In patients with symptoms (arrhythmic syncope/VT) and BB is ineffective/not tolerated and LCSD or other drugs are not effective alternatives		
I LOE C		In patients with symptoms (arrhythmic syncope/not tolerated VT) while receiving BB and genotype-specific therapy	ESC: II a [13]
II a LOE C		In patients with symptoms if BB and genotype-specific therapy are not tolerated ot contraindicated	
II b LOE B		In asymptomatic high risk patients (1-2-3- LQTS risk calculator) in addition to genotype-specific therapy (mexiletine in LQTS3)	
II b LOE C	Clinical RF:		New (QTc > 500 ms was II b) [12,13]
	QTc > 550 ms,		
	QTc > 500 ms in LQTS1, in females LQTS2, in males LQTS3, and/or mutations of JLN, Timothy, calmodulinopathies		
III LOE C	In asymptomatic low-risk patients without BB therapy		
**SQTS**			
II a		In patients with arrhythmic syncope	NEW-ESC
**CPVT**			
II a LOE C	In patients with aborted SCD as initial presentation ICD is reasonable in association with drug therapy with or without LCSD. Drug therapy and/or LCSD without ICD may be an alternative	Arrhythmic syncope and/or documented bidirectional/polimorphic VT in therapy with BB and flecainide at maximal tolerated doses	I [12,13]
			ESC I [12,13,14]
II b LOE C	Bidirectional/polimorphic VT despite OMT, with or without LCSD		I [14]
**BRUGADA**			
II a	Type I spontaneous pattern and recent syncope due to probable VA (LOE B)	Type I pattern and arrhythmic or unexplained syncope (LOE C)	I [12]
II b LOE C	Drug induced type I pattern and recent syncope due to probable VA	Selected asymptomatic patients with inducible VF (1–2 extrastimuli at EPS)	New
III LOE C	In asymptomatic patients without RF		
**ERS**			
II b		ICD or quinidine in:	NEW-ESC
		-Patients with ERP and arrhythmic syncope and additional RF (family history of unexplained SD < 40 yrs, family history of ERS)	
		-Asymptomatic subjects with high risk ERP (J waves > 2 mm, dynamic changes of J point and ST morphology) in the presence of family history of unexplained juvenile SCD	
**CHD**			
I LOE C		ACHD with biventricular physiology, systemic LV with NYHA II-III, EF ≤ 35% after ≥ 3 months of OMT.	
II a LOE C	Systemic LV EF < 35% and SVT or presumed syncope due to arrhythmias	Presumed syncope due to arrhythmias with either moderate (at least) ventricular dysfunction or EPS inducible SMVT	
II a LOE C		TOF: arrhythmia symptoms and positive EPS, or combinations of RF (moderate RV or LV dysfunction, extensive RV scarring at CMR, QRS ≥ 180 ms, severe QRS fragmentation)	
II b LOE C	Spontaneous hemodynamically stable SVT after hemodynamic/EPS evaluation. Ablation or surgery may be alternatives is selected cases		New
II b LOE C	Unexplained syncope in presence of ventricular dysfunction, NS VT, inducible VA at EPS		
II b LOE C	Single or systemic RV with EF ≤ 35%, especially with additional RF: VT, arrhythmic syncope, severe systemic AV valve regurgitation	Advanced dysfunction of single ventricle or systemic RV and additional RF: NSVT, NYHA II-III, severe AV valve regurgitation, QRS ≥ 140 ms (TGA)	

ACHD: adults with CHD, ACM: arryhtmogenic cardiomyopathy, BB: beta blocker, CHD: congenital heart disease, CHF: congestive heart failure, CMR: cardiac magnetic resonance, DCM: dilated cardiomyopathy; EF: ejection fraction, EPS: electrophysiologic study, ERP: early repolarization pattern, ERS: early repolarization syndrome, FLMC: filamin C mutation, HCM: hyperthrophic cardiomyopathy; JLN: Jerwell Lange Nielsen, LCSD: left cardiac sympathetic denervation, LGE: late gadolinium enhancement, LMNA: lamin A mutation, LV: left ventricular, LVH: LV hyperthrophy, NSVT: non-sustained VT, OMT: optimized medical therapy, PLN: phospholamban mutation, RBM20: RNA-binding motif protein 20 mutation, RF: risk factors, SCD: sudden cardiac death, SMVT: sustained monomorphic VT, SVT: sustained VT, TGA: transposition of the great arteries, TOF: tetralogy of Fallot after repair, VA: ventricular arrhythmia, VF: ventricular fibrillation, VT: ventricular tachycardia.

**Table 4:** Summary of recommended pacemaker (in case of AVB) and ICD implantation access and pacing mode according to age and weight of paediatric patients. See text for further details.

PACEMAKER		
AGE, WEIGHT	PACING SYSTEM ACCESS	PACING MODE
Neonates, infants, children 0–15 kg	Epicardial	VVI/VVIR/DDD
Children > 15 kg	Transvenous	VVIR
Adolescents (post-puberty)	Transvenous	DDD
ICD		
AGE, WEIGHT	PACING SYSTEM ACCESS	PACING MODE
Infants, Children (<30 kg)	Epicardial + coils	VVI
Children>30 kg	Transvenous	VVI
Adolescents	Transvenous	VVI-DDD
Adolescents	S-ICD	

## Data Availability

There are not research data.
